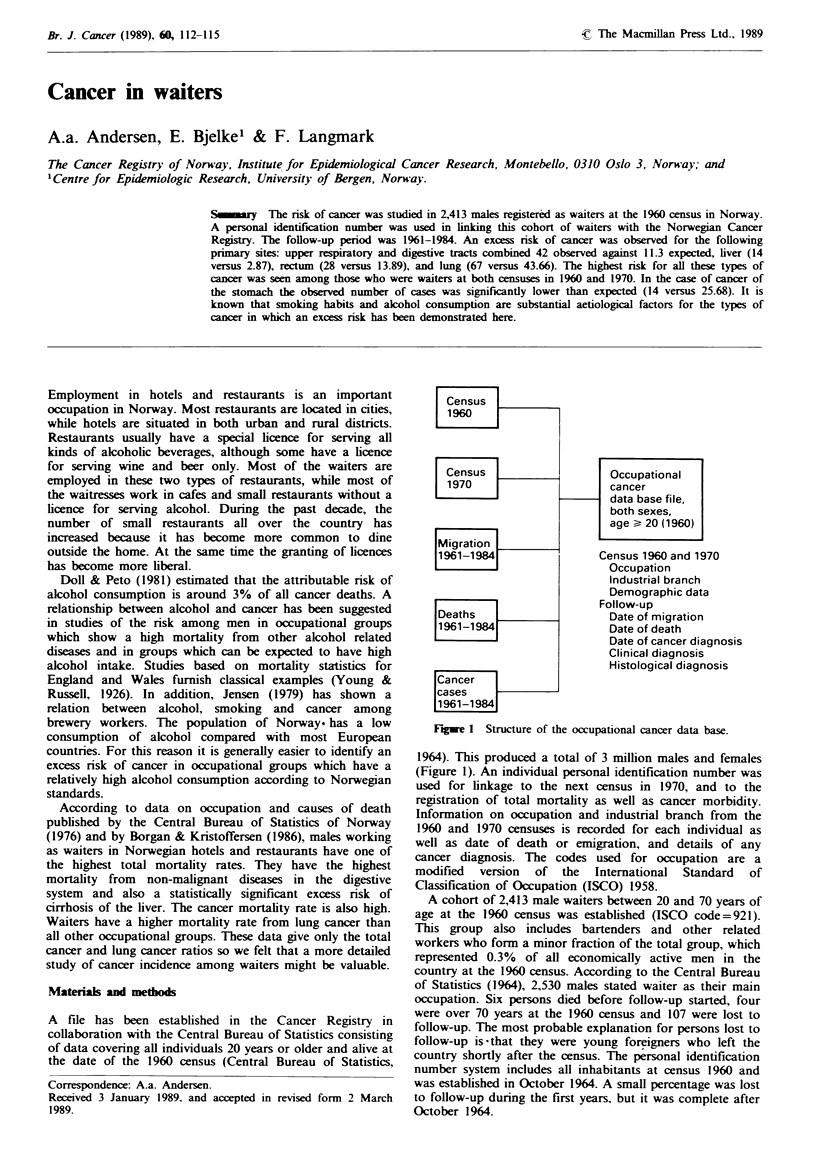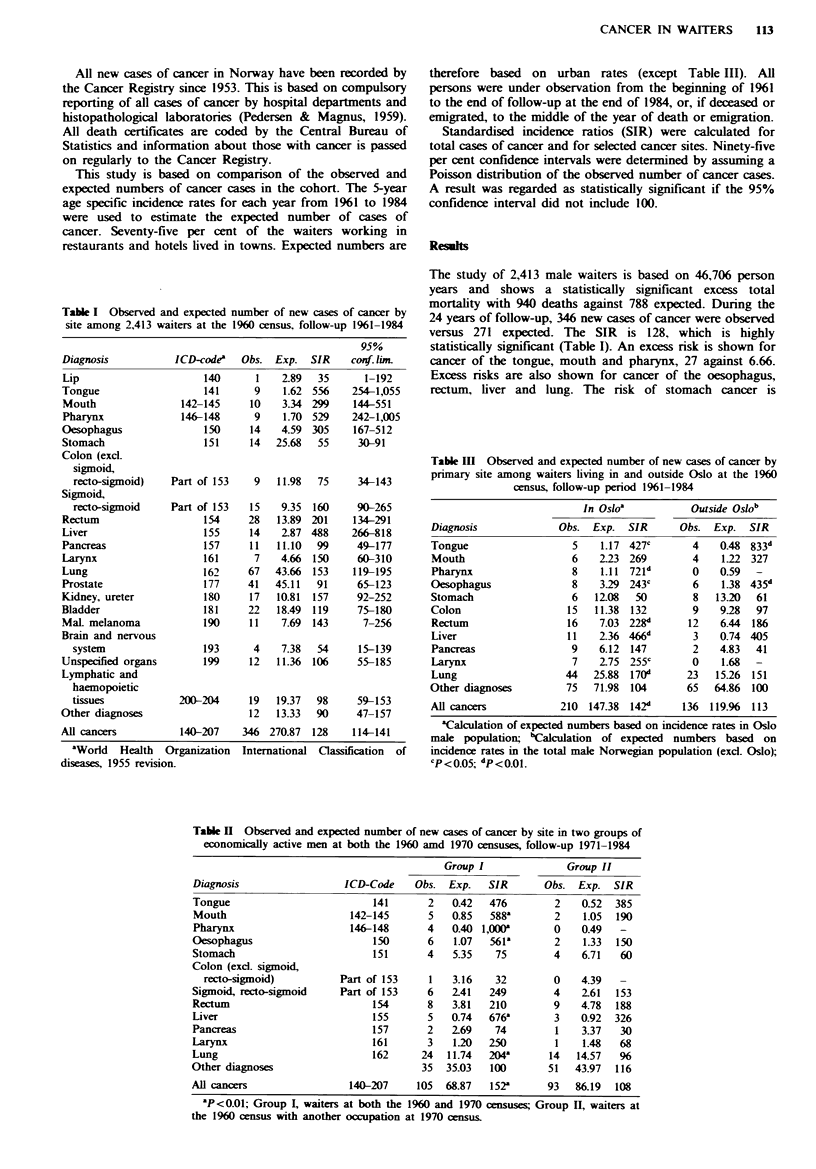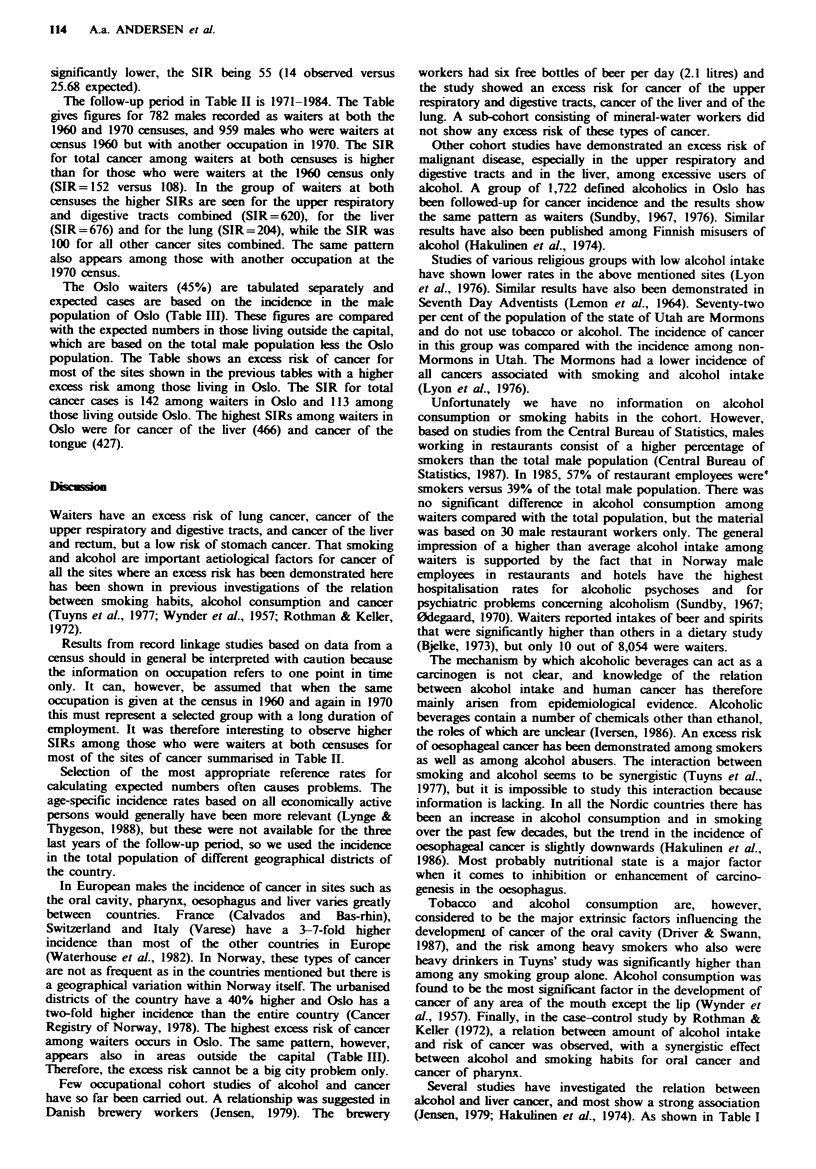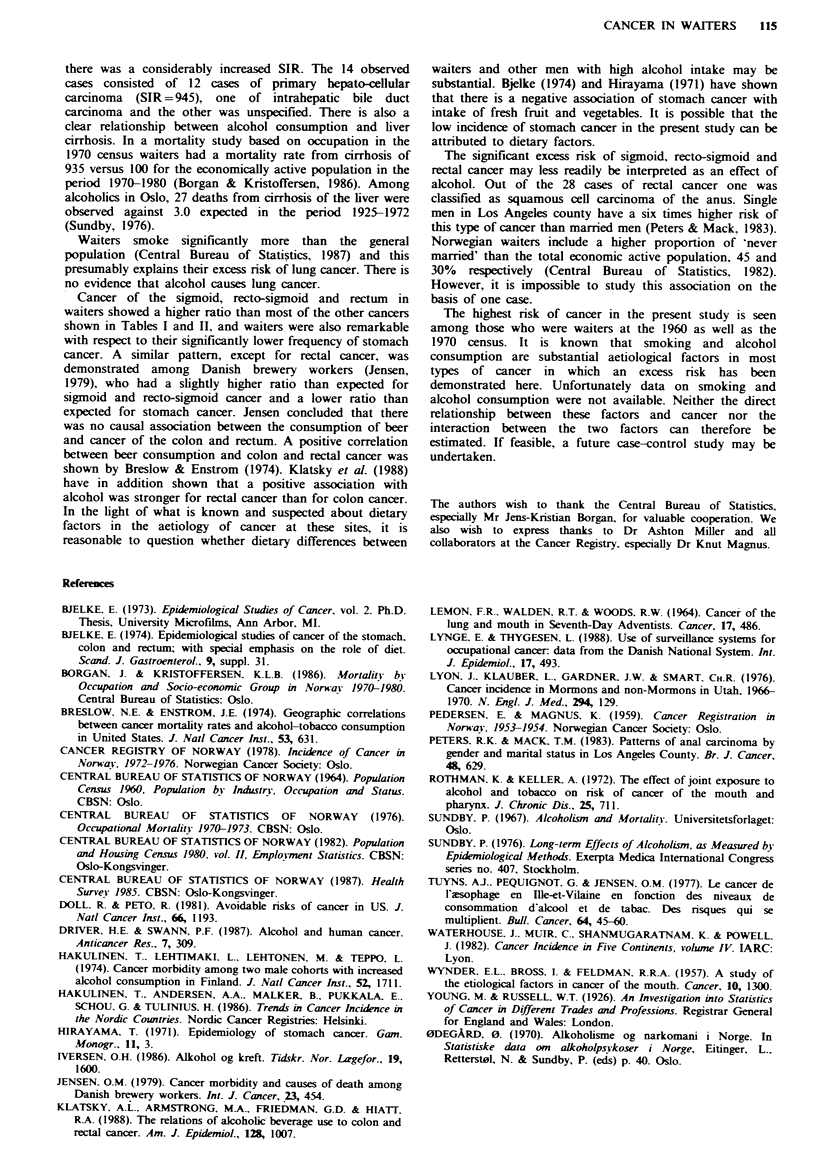# Cancer in waiters.

**DOI:** 10.1038/bjc.1989.232

**Published:** 1989-07

**Authors:** A. Andersen, E. Bjelke, F. Langmark

**Affiliations:** Cancer Registry of Norway, Institute for Epidemiological Cancer Research, Oslo.

## Abstract

The risk of cancer was studied in 2,413 males registered as waiters at the 1960 census in Norway. A personal identification number was used in linking this cohort of waiters with the Norwegian Cancer Registry. The follow-up period was 1961-1984. An excess risk of cancer was observed for the following primary sites: upper respiratory and digestive tracts combined 42 observed against 11.3 expected, liver (14 versus 2.87), rectum (28 versus 13.89), and lung (67 versus 43.66). The highest risk for all these types of cancer was seen among those who were waiters at both censuses in 1960 and 1970. In the case of cancer of the stomach the observed number of cases was significantly lower than expected (14 versus 25.68). It is known that smoking habits and alcohol consumption are substantial aetiological factors for the types of cancer in which an excess risk has been demonstrated here.


*Br. J. Cancer* (1989), **60**, 112–115 © The Macmillan Press Ltd., 1989

### Cancer in waiters

A.a. Andersen, E. Bjelke[1] & F. Langmark

*The Cancer Registry of Norway, Institute for Epidemiological Cancer Research, Montebello, 0310 Oslo 3, Norway; and [1]Centre for Epidemiologic Research, University of Bergen, Norway.*

**Summary** The risk of cancer was studied in 2,413 males registered as waiters at the 1960 census in Norway. A personal identification number was used in linking this cohort of waiters with the Norwegian Cancer Registry. The follow-up period was 1961–1984. An excess risk of cancer was observed for the following primary sites: upper respiratory and digestive tracts combined 42 observed against 11.3 expected, liver (14 versus 2.87), rectum (28 versus 13.89), and lung (67 versus 43.66). The highest risk for all these types of cancer was seen among those who were waiters at both censuses in 1960 and 1970. In the case of cancer of the stomach the observed number of cases was significantly lower than expected (14 versus 25.68). It is known that smoking habits and alcohol consumption are substantial aetiological factors for the types of cancer in which an excess risk has been demonstrated here.

Employment in hotels and restaurants is an important occupation in Norway. Most restaurants are located in cities, while hotels are situated in both urban and rural districts. Restaurants usually have a special licence for serving all kinds of alcoholic beverages, although some have a licence for serving wine and beer only. Most of the waiters are employed in these two types of restaurants, while most of the waitresses work in cafes and small restaurants without a licence for serving alcohol. During the past decade, the number of small restaurants all over the country has increased because it has become more common to dine outside the home. At the same time the granting of licences has become more liberal.

Doll & Peto (1981) estimated that the attributable risk of alcohol consumption is around 3% of all cancer deaths. A relationship between alcohol and cancer has been suggested in studies of the risk among men in occupational groups which show a high mortality from other alcohol related diseases and in groups which can be expected to have high alcohol intake. Studies based on mortality statistics for England and Wales furnish classical examples (Young & Russell, 1926). In addition, Jensen (1979) has shown a relation between alcohol, smoking and cancer among brewery workers. The population of Norway has a low consumption of alcohol compared with most European countries. For this reason it is generally easier to identify an excess risk of cancer in occupational groups which have a relatively high alcohol consumption according to Norwegian standards.

According to data on occupation and causes of death published by the Central Bureau of Statistics of Norway (1976) and by Borgan & Kristoffersen (1986), males working as waiters in Norwegian hotels and restaurants have one of the highest total mortality rates. They have the highest mortality from non-malignant diseases in the digestive system and also a statistically significant excess risk of cirrhosis of the liver. The cancer mortality rate is also high. Waiters have a higher mortality rate from lung cancer than all other occupational groups. These data give only the total cancer and lung cancer ratios so we felt that a more detailed study of cancer incidence among waiters might be valuable.

### Materials and methods

A file has been established in the Cancer Registry in collaboration with the Central Bureau of Statistics consisting of data covering all individuals 20 years or older and alive at the date of the 1960 census (Central Bureau of Statistics,

Correspondence: A.a. Andersen.
Received 3 January 1989, and accepted in revised form 2 March 1989.

**Figure 1** Structure of the occupational cancer data base.

1964). This produced a total of 3 million males and females (Figure 1). An individual personal identification number was used for linkage to the next census in 1970, and to the registration of total mortality as well as cancer morbidity. Information on occupation and industrial branch from the 1960 and 1970 censuses is recorded for each individual as well as date of death or emigration, and details of any cancer diagnosis. The codes used for occupation are a modified version of the International Standard of Classification of Occupation (ISCO) 1958.

A cohort of 2,413 male waiters between 20 and 70 years of age at the 1960 census was established (ISCO code = 921). This group also includes bartenders and other related workers who form a minor fraction of the total group, which represented 0.3% of all economically active men in the country at the 1960 census. According to the Central Bureau of Statistics (1964), 2,530 males stated waiter as their main occupation. Six persons died before follow-up started, four were over 70 years at the 1960 census and 107 were lost to follow-up. The most probable explanation for persons lost to follow-up is that they were young foreigners who left the country shortly after the census. The personal identification number system includes all inhabitants at census 1960 and was established in October 1964. A small percentage was lost to follow-up during the first years, but it was complete after October 1964.

All new cases of cancer in Norway have been recorded by the Cancer Registry since 1953. This is based on compulsory reporting of all cases of cancer by hospital departments and histopathological laboratories (Pedersen & Magnus, 1959). All death certificates are coded by the Central Bureau of Statistics and information about those with cancer is passed on regularly to the Cancer Registry.

This study is based on comparison of the observed and expected numbers of cancer cases in the cohort. The 5-year age specific incidence rates for each year from 1961 to 1984 were used to estimate the expected number of cases of cancer. Seventy-five per cent of the waiters working in restaurants and hotels lived in towns. Expected numbers are therefore based on urban rates (except Table III). All persons were under observation from the beginning of 1961 to the end of follow-up at the end of 1984, or, if deceased or emigrated, to the middle of the year of death or emigration.

Standardised incidence ratios (SIR) were calculated for total cases of cancer and for selected cancer sites. Ninety-five per cent confidence intervals were determined by assuming a Poisson distribution of the observed number of cancer cases. A result was regarded as statistically significant if the 95% confidence interval did not include 100.

**Table I** Observed and expected number of new cases of cancer by site among 2,413 waiters at the 1960 census, follow-up 1961–1984

| *Diagnosis* | *ICD-code*[a] | *Obs.* | *Exp.* | *SIR* | *95% conf. lim.* |
|---|---|---|---|---|---|
| Lip | 140 | 1 | 2.89 | 35 | 1–192 |
| Tongue | 141 | 9 | 1.62 | 556 | 254–1,055 |
| Mouth | 142–145 | 10 | 3.34 | 299 | 144–551 |
| Pharynx | 146–148 | 9 | 1.70 | 529 | 242–1,005 |
| Oesophagus | 150 | 14 | 4.59 | 305 | 167–512 |
| Stomach | 151 | 14 | 25.68 | 55 | 30–91 |
| Colon (excl. sigmoid, recto-sigmoid) | Part of 153 | 9 | 11.98 | 75 | 34–143 |
| Sigmoid, recto-sigmoid | Part of 153 | 15 | 9.35 | 160 | 90–265 |
| Rectum | 154 | 28 | 13.89 | 201 | 134–291 |
| Liver | 155 | 14 | 2.87 | 488 | 266–818 |
| Pancreas | 157 | 11 | 11.10 | 99 | 49–177 |
| Larynx | 161 | 7 | 4.66 | 150 | 60–310 |
| Lung | 162 | 67 | 43.66 | 153 | 119–195 |
| Prostate | 177 | 41 | 45.11 | 91 | 65–123 |
| Kidney, ureter | 180 | 17 | 10.81 | 157 | 92–252 |
| Bladder | 181 | 22 | 18.49 | 119 | 75–180 |
| Mal. melanoma | 190 | 11 | 7.69 | 143 | 7–256 |
| Brain and nervous system | 193 | 4 | 7.38 | 54 | 15–139 |
| Unspecified organs | 199 | 12 | 11.36 | 106 | 55–185 |
| Lymphatic and haemopoietic tissues | 200–204 | 19 | 19.37 | 98 | 59–153 |
| Other diagnoses | | 12 | 13.33 | 90 | 47–157 |
| All cancers | 140–207 | 346 | 270.87 | 128 | 114–141 |

[a]World Health Organization International Classification of diseases, 1955 revision.

### Results

The study of 2,413 male waiters is based on 46,706 person years and shows a statistically significant excess total mortality with 940 deaths against 788 expected. During the 24 years of follow-up, 346 new cases of cancer were observed versus 271 expected. The SIR is 128, which is highly statistically significant (Table I). An excess risk is shown for cancer of the tongue, mouth and pharynx, 27 against 6.66. Excess risks are also shown for cancer of the oesophagus, rectum, liver and lung. The risk of stomach cancer is

**Table III** Observed and expected number of new cases of cancer by primary site among waiters living in and outside Oslo at the 1960 census, follow-up period 1961–1984

| | *In Oslo*[a] | | | *Outside Oslo*[b] | | |
|---|---|---|---|---|---|---|
| *Diagnosis* | *Obs.* | *Exp.* | *SIR* | *Obs.* | *Exp.* | *SIR* |
| Tongue | 5 | 1.17 | 427[c] | 4 | 0.48 | 833[d] |
| Mouth | 6 | 2.23 | 269 | 4 | 1.22 | 327 |
| Pharynx | 8 | 1.11 | 721[d] | 0 | 0.59 | – |
| Oesophagus | 8 | 3.29 | 243[c] | 6 | 1.38 | 435[d] |
| Stomach | 6 | 12.08 | 50 | 8 | 13.20 | 61 |
| Colon | 15 | 11.38 | 132 | 9 | 9.28 | 97 |
| Rectum | 16 | 7.03 | 228[d] | 12 | 6.44 | 186 |
| Liver | 11 | 2.36 | 466[d] | 3 | 0.74 | 405 |
| Pancreas | 9 | 6.12 | 147 | 2 | 4.83 | 41 |
| Larynx | 7 | 2.75 | 255[c] | 0 | 1.68 | – |
| Lung | 44 | 25.88 | 170[d] | 23 | 15.26 | 151 |
| Other diagnoses | 75 | 71.98 | 104 | 65 | 64.86 | 100 |
| All cancers | 210 | 147.38 | 142[d] | 136 | 119.96 | 113 |

[a]Calculation of expected numbers based on incidence rates in Oslo male population; [b]Calculation of expected numbers based on incidence rates in the total male Norwegian population (excl. Oslo); [c]$P<0.05$; [d]$P<0.01$.

**Table II** Observed and expected number of new cases of cancer by site in two groups of economically active men at both the 1960 amd 1970 censuses, follow-up 1971–1984

| | | *Group I* | | | *Group II* | | |
|---|---|---|---|---|---|---|---|
| *Diagnosis* | *ICD-Code* | *Obs.* | *Exp.* | *SIR* | *Obs.* | *Exp.* | *SIR* |
| Tongue | 141 | 2 | 0.42 | 476 | 2 | 0.52 | 385 |
| Mouth | 142–145 | 5 | 0.85 | 588[a] | 2 | 1.05 | 190 |
| Pharynx | 146–148 | 4 | 0.40 | 1,000[a] | 0 | 0.49 | – |
| Oesophagus | 150 | 6 | 1.07 | 561[a] | 2 | 1.33 | 150 |
| Stomach | 151 | 4 | 5.35 | 75 | 4 | 6.71 | 60 |
| Colon (excl. sigmoid, recto-sigmoid) | Part of 153 | 1 | 3.16 | 32 | 0 | 4.39 | – |
| Sigmoid, recto-sigmoid | Part of 153 | 6 | 2.41 | 249 | 4 | 2.61 | 153 |
| Rectum | 154 | 8 | 3.81 | 210 | 9 | 4.78 | 188 |
| Liver | 155 | 5 | 0.74 | 676[a] | 3 | 0.92 | 326 |
| Pancreas | 157 | 2 | 2.69 | 74 | 1 | 3.37 | 30 |
| Larynx | 161 | 3 | 1.20 | 250 | 1 | 1.48 | 68 |
| Lung | 162 | 24 | 11.74 | 204[a] | 14 | 14.57 | 96 |
| Other diagnoses | | 35 | 35.03 | 100 | 51 | 43.97 | 116 |
| All cancers | 140–207 | 105 | 68.87 | 152[a] | 93 | 86.19 | 108 |

[a]$P<0.01$; Group I, waiters at both the 1960 and 1970 censuses; Group II, waiters at the 1960 census with another occupation at 1970 census.

significantly lower, the SIR being 55 (14 observed versus 25.68 expected).

The follow-up period in Table II is 1971–1984. The Table gives figures for 782 males recorded as waiters at both the 1960 and 1970 censuses, and 959 males who were waiters at census 1960 but with another occupation in 1970. The SIR for total cancer among waiters at both censuses is higher than for those who were waiters at the 1960 census only (SIR = 152 versus 108). In the group of waiters at both censuses the higher SIRs are seen for the upper respiratory and digestive tracts combined (SIR = 620), for the liver (SIR = 676) and for the lung (SIR = 204), while the SIR was 100 for all other cancer sites combined. The same pattern also appears among those with another occupation at the 1970 census.

The Oslo waiters (45%) are tabulated separately and expected cases are based on the incidence in the male population of Oslo (Table III). These figures are compared with the expected numbers in those living outside the capital, which are based on the total male population less the Oslo population. The Table shows an excess risk of cancer for most of the sites shown in the previous tables with a higher excess risk among those living in Oslo. The SIR for total cancer cases is 142 among waiters in Oslo and 113 among those living outside Oslo. The highest SIRs among waiters in Oslo were for cancer of the liver (466) and cancer of the tongue (427).

### Discussion

Waiters have an excess risk of lung cancer, cancer of the upper respiratory and digestive tracts, and cancer of the liver and rectum, but a low risk of stomach cancer. That smoking and alcohol are important aetiological factors for cancer of all the sites where an excess risk has been demonstrated here has been shown in previous investigations of the relation between smoking habits, alcohol consumption and cancer (Tuyns et al., 1977; Wynder et al., 1957; Rothman & Keller, 1972).

Results from record linkage studies based on data from a census should in general be interpreted with caution because the information on occupation refers to one point in time only. It can, however, be assumed that when the same occupation is given at the census in 1960 and again in 1970 this must represent a selected group with a long duration of employment. It was therefore interesting to observe higher SIRs among those who were waiters at both censuses for most of the sites of cancer summarised in Table II.

Selection of the most appropriate reference rates for calculating expected numbers often causes problems. The age-specific incidence rates based on all economically active persons would generally have been more relevant (Lynge & Thygeson, 1988), but these were not available for the three last years of the follow-up period, so we used the incidence in the total population of different geographical districts of the country.

In European males the incidence of cancer in sites such as the oral cavity, pharynx, oesophagus and liver varies greatly between countries. France (Calvados and Bas-rhin), Switzerland and Italy (Varese) have a 3–7-fold higher incidence than most of the other countries in Europe (Waterhouse et al., 1982). In Norway, these types of cancer are not as frequent as in the countries mentioned but there is a geographical variation within Norway itself. The urbanised districts of the country have a 40% higher and Oslo has a two-fold higher incidence than the entire country (Cancer Registry of Norway, 1978). The highest excess risk of cancer among waiters occurs in Oslo. The same pattern, however, appears also in areas outside the capital (Table III). Therefore, the excess risk cannot be a big city problem only.

Few occupational cohort studies of alcohol and cancer have so far been carried out. A relationship was suggested in Danish brewery workers (Jensen, 1979). The brewery workers had six free bottles of beer per day (2.1 litres) and the study showed an excess risk for cancer of the upper respiratory and digestive tracts, cancer of the liver and of the lung. A sub-cohort consisting of mineral-water workers did not show any excess risk of these types of cancer.

Other cohort studies have demonstrated an excess risk of malignant disease, especially in the upper respiratory and digestive tracts and in the liver, among excessive users of alcohol. A group of 1,722 defined alcoholics in Oslo has been followed-up for cancer incidence and the results show the same pattern as waiters (Sundby, 1967, 1976). Similar results have also been published among Finnish misusers of alcohol (Hakulinen et al., 1974).

Studies of various religious groups with low alcohol intake have shown lower rates in the above mentioned sites (Lyon et al., 1976). Similar results have also been demonstrated in Seventh Day Adventists (Lemon et al., 1964). Seventy-two per cent of the population of the state of Utah are Mormons and do not use tobacco or alcohol. The incidence of cancer in this group was compared with the incidence among non-Mormons in Utah. The Mormons had a lower incidence of all cancers associated with smoking and alcohol intake (Lyon et al., 1976).

Unfortunately we have no information on alcohol consumption or smoking habits in the cohort. However, based on studies from the Central Bureau of Statistics, males working in restaurants consist of a higher percentage of smokers than the total male population (Central Bureau of Statistics, 1987). In 1985, 57% of restaurant employees were smokers versus 39% of the total male population. There was no significant difference in alcohol consumption among waiters compared with the total population, but the material was based on 30 male restaurant workers only. The general impression of a higher than average alcohol intake among waiters is supported by the fact that in Norway male employees in restaurants and hotels have the highest hospitalisation rates for alcoholic psychoses and for psychiatric problems concerning alcoholism (Sundby, 1967; Ødegaard, 1970). Waiters reported intakes of beer and spirits that were significantly higher than others in a dietary study (Bjelke, 1973), but only 10 out of 8,054 were waiters.

The mechanism by which alcoholic beverages can act as a carcinogen is not clear, and knowledge of the relation between alcohol intake and human cancer has therefore mainly arisen from epidemiological evidence. Alcoholic beverages contain a number of chemicals other than ethanol, the roles of which are unclear (Iversen, 1986). An excess risk of oesophageal cancer has been demonstrated among smokers as well as among alcohol abusers. The interaction between smoking and alcohol seems to be synergistic (Tuyns et al., 1977), but it is impossible to study this interaction because information is lacking. In all the Nordic countries there has been an increase in alcohol consumption and in smoking over the past few decades, but the trend in the incidence of oesophageal cancer is slightly downwards (Hakulinen et al., 1986). Most probably nutritional state is a major factor when it comes to inhibition or enhancement of carcinogenesis in the oesophagus.

Tobacco and alcohol consumption are, however, considered to be the major extrinsic factors influencing the development of cancer of the oral cavity (Driver & Swann, 1987), and the risk among heavy smokers who also were heavy drinkers in Tuyns' study was significantly higher than among any smoking group alone. Alcohol consumption was found to be the most significant factor in the development of cancer of any area of the mouth except the lip (Wynder et al., 1957). Finally, in the case–control study by Rothman & Keller (1972), a relation between amount of alcohol intake and risk of cancer was observed, with a synergistic effect between alcohol and smoking habits for oral cancer and cancer of pharynx.

Several studies have investigated the relation between alcohol and liver cancer, and most show a strong association (Jensen, 1979; Hakulinen et al., 1974). As shown in Table I

there was a considerably increased SIR. The 14 observed cases consisted of 12 cases of primary hepato-cellular carcinoma (SIR = 945), one of intrahepatic bile duct carcinoma and the other was unspecified. There is also a clear relationship between alcohol consumption and liver cirrhosis. In a mortality study based on occupation in the 1970 census waiters had a mortality rate from cirrhosis of 935 versus 100 for the economically active population in the period 1970–1980 (Borgan & Kristoffersen, 1986). Among alcoholics in Oslo, 27 deaths from cirrhosis of the liver were observed against 3.0 expected in the period 1925–1972 (Sundby, 1976).

Waiters smoke significantly more than the general population (Central Bureau of Statistics, 1987) and this presumably explains their excess risk of lung cancer. There is no evidence that alcohol causes lung cancer.

Cancer of the sigmoid, recto-sigmoid and rectum in waiters showed a higher ratio than most of the other cancers shown in Tables I and II, and waiters were also remarkable with respect to their significantly lower frequency of stomach cancer. A similar pattern, except for rectal cancer, was demonstrated among Danish brewery workers (Jensen, 1979), who had a slightly higher ratio than expected for sigmoid and recto-sigmoid cancer and a lower ratio than expected for stomach cancer. Jensen concluded that there was no causal association between the consumption of beer and cancer of the colon and rectum. A positive correlation between beer consumption and colon and rectal cancer was shown by Breslow & Enstrom (1974). Klatsky *et al.* (1988) have in addition shown that a positive association with alcohol was stronger for rectal cancer than for colon cancer. In the light of what is known and suspected about dietary factors in the aetiology of cancer at these sites, it is reasonable to question whether dietary differences between waiters and other men with high alcohol intake may be substantial. Bjelke (1974) and Hirayama (1971) have shown that there is a negative association of stomach cancer with intake of fresh fruit and vegetables. It is possible that the low incidence of stomach cancer in the present study can be attributed to dietary factors.

The significant excess risk of sigmoid, recto-sigmoid and rectal cancer may less readily be interpreted as an effect of alcohol. Out of the 28 cases of rectal cancer one was classified as squamous cell carcinoma of the anus. Single men in Los Angeles county have a six times higher risk of this type of cancer than married men (Peters & Mack, 1983). Norwegian waiters include a higher proportion of 'never married' than the total economic active population, 45 and 30% respectively (Central Bureau of Statistics, 1982). However, it is impossible to study this association on the basis of one case.

The highest risk of cancer in the present study is seen among those who were waiters at the 1960 as well as the 1970 census. It is known that smoking and alcohol consumption are substantial aetiological factors in most types of cancer in which an excess risk has been demonstrated here. Unfortunately data on smoking and alcohol consumption were not available. Neither the direct relationship between these factors and cancer nor the interaction between the two factors can therefore be estimated. If feasible, a future case–control study may be undertaken.

The authors wish to thank the Central Bureau of Statistics, especially Mr Jens-Kristian Borgan, for valuable cooperation. We also wish to express thanks to Dr Ashton Miller and all collaborators at the Cancer Registry, especially Dr Knut Magnus.

**References**

BJELKE, E. (1973). *Epidemiological Studies of Cancer*, vol. 2. Ph.D. Thesis, University Microfilms, Ann Arbor, MI.

BJELKE, E. (1974). Epidemiological studies of cancer of the stomach, colon and rectum; with special emphasis on the role of diet. *Scand. J. Gastroenterol.*, **9**, suppl. 31.

BORGAN, J. & KRISTOFFERSEN, K.L.B. (1986). *Mortality by Occupation and Socio-economic Group in Norway 1970–1980*. Central Bureau of Statistics: Oslo.

BRESLOW, N.E. & ENSTROM, J.E. (1974). Geographic correlations between cancer mortality rates and alcohol–tobacco consumption in United States. *J. Natl Cancer Inst.*, **53**, 631.

CANCER REGISTRY OF NORWAY (1978). *Incidence of Cancer in Norway, 1972–1976*. Norwegian Cancer Society: Oslo.

CENTRAL BUREAU OF STATISTICS OF NORWAY (1964). *Population Census 1960, Population by Industry, Occupation and Status*. CBSN: Oslo.

CENTRAL BUREAU OF STATISTICS OF NORWAY (1976). *Occupational Mortality 1970–1973*. CBSN: Oslo.

CENTRAL BUREAU OF STATISTICS OF NORWAY (1982). *Population and Housing Census 1980, vol. II, Employment Statistics*. CBSN: Oslo-Kongsvinger.

CENTRAL BUREAU OF STATISTICS OF NORWAY (1987). *Health Survey 1985*. CBSN: Oslo-Kongsvinger.

DOLL, R. & PETO, R. (1981). Avoidable risks of cancer in US. *J. Natl Cancer Inst.*, **66**, 1193.

DRIVER, H.E. & SWANN, P.F. (1987). Alcohol and human cancer. *Anticancer Res.*, **7**, 309.

HAKULINEN, T., LEHTIMAKI, L., LEHTONEN, M. & TEPPO, L. (1974). Cancer morbidity among two male cohorts with increased alcohol consumption in Finland. *J. Natl Cancer Inst.*, **52**, 1711.

HAKULINEN, T., ANDERSEN, A.A., MALKER, B., PUKKALA, E., SCHOU, G. & TULINIUS, H. (1986). *Trends in Cancer Incidence in the Nordic Countries*. Nordic Cancer Registries: Helsinki.

HIRAYAMA, T. (1971). Epidemiology of stomach cancer. *Gann. Monogr.*, **11**, 3.

IVERSEN, O.H. (1986). Alkohol og kreft. *Tidskr. Nor. Lægefor.*, **19**, 1600.

JENSEN, O.M. (1979). Cancer morbidity and causes of death among Danish brewery workers. *Int. J. Cancer*, **23**, 454.

KLATSKY, A.L., ARMSTRONG, M.A., FRIEDMAN, G.D. & HIATT, R.A. (1988). The relations of alcoholic beverage use to colon and rectal cancer. *Am. J. Epidemiol.*, **128**, 1007.

LEMON, F.R., WALDEN, R.T. & WOODS, R.W. (1964). Cancer of the lung and mouth in Seventh-Day Adventists. *Cancer*, **17**, 486.

LYNGE, E. & THYGESEN, L. (1988). Use of surveillance systems for occupational cancer: data from the Danish National System. *Int. J. Epidemiol.*, **17**, 493.

LYON, J., KLAUBER, L., GARDNER, J.W. & SMART, CH.R. (1976). Cancer incidence in Mormons and non-Mormons in Utah, 1966–1970. *N. Engl. J. Med.*, **294**, 129.

PEDERSEN, E. & MAGNUS, K. (1959). *Cancer Registration in Norway, 1953–1954*. Norwegian Cancer Society: Oslo.

PETERS, R.K. & MACK, T.M. (1983). Patterns of anal carcinoma by gender and marital status in Los Angeles County. *Br. J. Cancer*, **48**, 629.

ROTHMAN, K. & KELLER, A. (1972). The effect of joint exposure to alcohol and tobacco on risk of cancer of the mouth and pharynx. *J. Chronic Dis.*, **25**, 711.

SUNDBY, P. (1967). *Alcoholism and Mortality*. Universitetsforlaget: Oslo.

SUNDBY, P. (1976). *Long-term Effects of Alcoholism, as Measured by Epidemiological Methods*. Exerpta Medica International Congress series no. 407, Stockholm.

TUYNS, A.J., PEQUIGNOT, G. & JENSEN, O.M. (1977). Le cancer de l'æsophage en Ille-et-Vilaine en fonction des niveaux de consommation d'alcool et de tabac. Des risques qui se multiplient. *Bull. Cancer*, **64**, 45–60.

WATERHOUSE, J., MUIR, C., SHANMUGARATNAM, K. & POWELL, J. (1982). *Cancer Incidence in Five Continents, volume IV*. IARC: Lyon.

WYNDER, E.L., BROSS, I. & FELDMAN, R.R.A. (1957). A study of the etiological factors in cancer of the mouth. *Cancer*, **10**, 1300.

YOUNG, M. & RUSSELL, W.T. (1926). *An Investigation into Statistics of Cancer in Different Trades and Professions*. Registrar General for England and Wales: London.

ØDEGÅRD, Ø. (1970). Alkoholisme og narkomani i Norge. In *Statistiske data om alkoholpsykoser i Norge*, Eitinger, L., Retterstøl, N. & Sundby, P. (eds) p. 40. Oslo.